# Stereotactic laser interstitial thermal therapy corpus callosotomy for the treatment of pediatric drug‐resistant epilepsy

**DOI:** 10.1002/epi4.12559

**Published:** 2021-11-24

**Authors:** Arka N. Mallela, Jasmine L. Hect, Hussam Abou‐Al‐Shaar, Emefa Akwayena, Taylor J. Abel

**Affiliations:** ^1^ Department of Neurological Surgery University of Pittsburgh Pittsburgh PA USA; ^2^ Department of Bioengineering University of Pittsburgh Pittsburgh PA USA

**Keywords:** corpus callosotomy, drop attacks, epilepsy, MRgLITT, MRI‐guided laser interstitial thermal therapy

## Abstract

**Objective:**

Corpus callosotomy is a safe and effective procedure for reducing the frequency of drop attacks. MR‐guided laser interstitial thermal therapy (MRgLITT) offers a minimally invasive alternative to conventional open craniotomy for callosotomy. We hypothesized that MRgLITT callosotomy could be safely performed in pediatric patients with similar seizure control.

**Methods:**

We present an institutional case series of 11 procedures in 10 patients for the treatment of drop attacks in drug‐refractory primary generalized epilepsy. MRgLITT was used for complete callosotomy, anterior two‐thirds, posterior, or ablation of residual callosal fibers following prior callosotomy (open or MRgLITT). We retrospectively reviewed clinical course, operative details, radiographic imaging, clinical outcomes, and complications.

**Results:**

Operative time ranged from 4‐8 hours, and median hospitalization was 2 days. No complications were encountered. Among the 7 patients with at least 3 months of follow‐up, 71% experienced freedom from drop attacks at longest follow‐up and 57% of cases showed improvement in their other seizure semiologies as well (Engel Class II: 28%, Class III: 28%, Class IV: 43%).

**Significance:**

MR‐guided LITT callosotomy is safe and effective modality in the management of pediatric patients with medically intractable epilepsy characterized by drop attacks. While this is among the largest pediatric series to date, further studies are required to delineate its safety and efficacy among such patients.

**Key points**

- MRgLITT offers a minimally invasive alternative to open corpus callosotomy in pediatric patients with drug‐resistant epilepsy.
- MRgLITT callosotomy resulted in shorter hospitalization length, no complications, and good response of drop attacks.
- MRgLITT is a safe and effective approach for performing corpus callosotomy.

## 1 INTRODUCTION

Corpus callosotomy is an effective palliative treatment for patients with generalized or multifocal drug‐resistant epilepsy suffering from potentially injurious drop attacks, who are not candidates for focal resection.^1^, ^2^ Drop attacks are extremely debilitating and frequently reduce quality of life^3^ and can cause repetitive mechanical injuries, such as head trauma. Partial or complete callosal disconnection interrupts the interhemispheric propagation of epileptogenic activity and is an effective technique for reducing drop attack frequency with relatively low morbidity.^1^, ^4^, ^5^ Open and endoscopic approaches for callosotomy in patients with drug‐resistant epilepsy have demonstrated drop attack freedom in 35%‐90% of patients and at least a 50% reduction in 74%‐100% of patients.^1^, ^4^, ^6^, ^7^, ^8^, ^9^

MRI‐guided laser interstitial thermal therapy (MRgLITT) is an emerging technique that offers minimally invasive modality for corpus callosum ablation.^10^, ^11^ MRgLITT offers significant advantages to open callosotomy, including its minimally invasive nature, shorter hospitalization and operative time, decreased total blood loss, and comparable seizure control rates.^11^, ^12^, ^13^ Various studies have demonstrated LITT callosotomy as an effective therapy for reducing seizure rates in adult^12^, ^14^, ^15^, ^16^, ^17^, ^18^, ^19^, ^20^, ^21^, ^22^ and pediatric^11^, ^16^, ^23^, ^24^ patients with drug‐resistant epilepsy, with comparable effectiveness to open callosotomy.^11^ This study presents experience performing MRgLITT callosotomy for the treatment of drug‐resistant epilepsy characterized by drop attacks in pediatric patients at a tertiary academic center. We provide supporting evidence that MRgLITT is feasible in pediatric populations and effective in reducing seizures, primarily drop attacks.

## 2 METHODS

### 2.1 Patient selection

A retrospective review of pediatric patients with drop attacks managed at Children's Hospital of Pittsburgh from January 2020 to October 2021 was performed. Institutional Review Board (IRB) approval was obtained as part of the global protocol for retrospective data collection in epilepsy patients. Patients were referred for callosotomy after evaluation by a multidisciplinary epilepsy conference in the case of drug‐resistant drop attacks. All patients underwent preoperative electroencephalography (EEG), adequate trials of at least two anti‐seizure medications, and neuropsychological evaluation. All procedures were performed by the senior author using the technique described below.

### 2.2 Preoperative imaging and LITT planning

All patients underwent preoperative epilepsy protocol MRI including volumetric T1 FSPGR with and without contrast, volumetric fluid attenuation inversion recovery (FLAIR), high‐definition fiber tractography (HDFT), resting‐state functional MRI, and arterial spin labeling. Intraoperative MRI imaging is acquired on 3T GE Discovery 750W, using the Monteris Medical NeuroBlate^®^ System (v26.0). Localizer and T1 FSPGR sequences (TR 27 msec, TE 19.1 msec, 5 mm slice thickness, acquisition time 8.2 seconds) are obtained to set thermography field of view and confirm electrode positioning prior to ablation of each target. MR thermography during ablation is acquired in the form of a gradient recalled echo (GRE) sequence (NeuroBlate^®^).

### 2.3 Surgical technique

**FIGURE 1:**
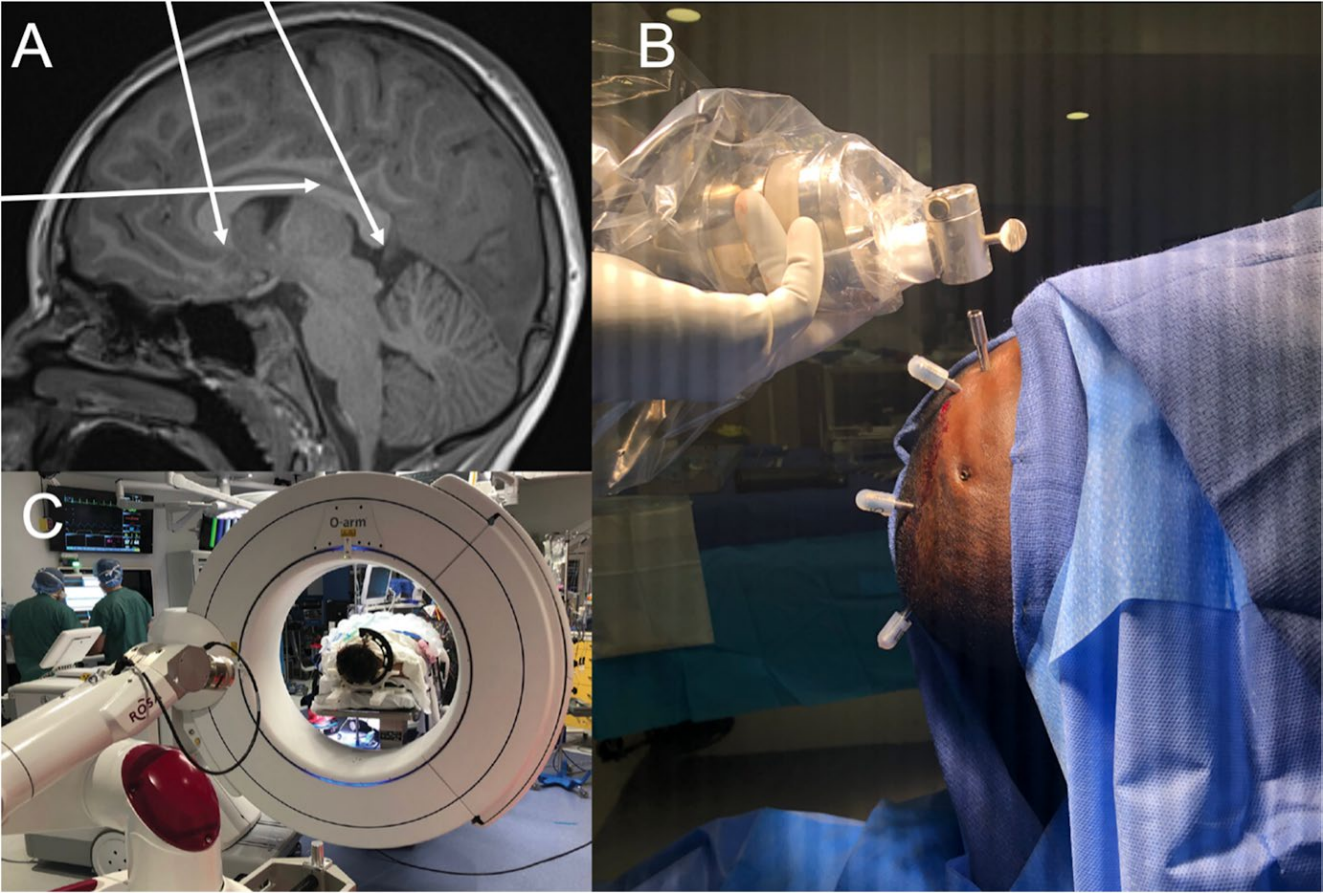
A, Example of a preoperative planning photo using T1‐weighted MRI to visualize LITT catheter trajectory to target the genu, isthmus, and splenium of the corpus callosum. B, Example of capped intraoperative electrode catheters. C, ROSA robot and O‐arm intraoperative imaging system. Not visualized is the Leksell stereotactic system head frame, which is preferred for LITT callosotomy for its flexibility

On the day of surgery, patients are brought to the operating room. High‐dose dexamethasone is given for anticipated cerebral edema and tapered over 1‐2 weeks postoperatively. Anti‐seizure medications are maintained. The patient's head is fixed in the Monteris Atma board or Leksell head frame. Bone fiducial registration is performed utilizing an intraoperative CT scan (O‐Arm). RMS of 0.8mm after registration is considered acceptable (see Figure 1).

The CT image is then merged with the pre‐operatively planned ablation trajectories using bone fiducial registration. The ROSA robot is then used to navigate to each trajectory, where trajectory guidance bolts (Monteris) are then placed at each of the entry sites. Care is taken to maintain normothermia throughout the procedure as hypothermia can make ablation more difficult. Once in the MRI room, accessed through a shielded door connecting the two rooms, an ablation probe is inserted through the guidance bolt along the planned trajectory and MRgLITT is performed under direct MR thermography. The ROSA robot navigates to each subsequent trajectory, and this process is repeated for each trajectory. Imaging is obtained to assess tissue changes and successful ablation of fibers (post‐contrast MPRAGE, T1, and DWI). Once acceptable ablation has been achieved, the patient is taken to OR where ablation bolts are removed and sutured.

Patients are admitted to the pediatric ICU for neuromonitoring. Patients may be discharged as early as day 1 and are not asked to adhere to any specific activity restrictions. No additional imaging is obtained until 3 months postoperative follow‐up.

### 2.4 Data collection

Data were collected in a retrospective fashion, including patient demographics and patient medical history, operative variables, and postoperative course. Patient outcomes included changes in frequency of drop attacks, change in frequency of all seizures, and postoperative neurological deficits. Pre‐ and postoperative imaging were reviewed to assess for the efficacy of disconnection and postoperative complications. Volume of ablated tissue was retrospectively calculated using Monteris software and intraoperative thermography sequences.

### 2.5 Statistics/data analysis

Descriptive statistics were exclusively used to present data in this small cohort. Continuous variables are presented as mean ± standard deviation, unless otherwise specified. Statistical analysis was conducted utilizing R (R Foundation for Statistical Computing, Vienna, Austria).

## 3 RESULTS

### 3.1 Cohort description

A total of 10 pediatric patients, ages 4‐21 years (13.5 ± 5.7), underwent 11 MRgLITT callosotomy procedures for the treatment of drug‐refractory primary generalized epilepsy with drop attacks at our institution from 2020 to 2021. Six of 10 patients were female. The patients were average 2 years old at initial diagnosis and had epilepsy for 10.9 ± 5 years prior to MRgLITT callosotomy. Patients presented with neurocognitive diagnoses of intellectual disability, ranging from mild to severe, including four with Lennox‐Gastaut syndrome, three with autism spectrum disorder, and one with tuberous sclerosis complex. Patients adhered to medical management, including levetiracetam (62.5% of patients), lamotrigine (50%), and oxcarbazepine (37.5%) (see Table 1).

**TABLE 1:** Cohort demographics and epilepsy history

	Summary	Case 1	Case 2	Case 3	Case 4	Case 5	Case 6	Case 7	Case 8	Case 9	Case 10	Case 11
Age (years), M (SD)	14.4 (5.1)	7	16	10	13	21	10	19	4	17	19	20
Female, n (%)	6 (55)	Female	Male	Male	Female	Male	Female	Male	Male	Female	Female	(Case 10)
Race, white, n (%)	7 (64)	White	White	White	White	African‐American	White	White	White	White	White	(Case 10)
Epilepsy (years), M (SD)	10.3 (4.9)	5	14	8	12.6	20.8	8	12	4	14	7	8
Epilepsy diagnosis		Generalized epilepsy, atonic	Focal seizures with impaired awareness, generalized atonic	Generalized myoclonic absence seizures, head drops	Generalized myoclonic atonic	Generalized myoclonic atonic	Generalized myoclonic atonic	Focal seizures with impaired awareness, generalized atonic	Focal to bilateral myoclonic‐tonic‐clonic, atonic	Generalized myoclonic‐tonic‐clonic, myoclonic	Focal epilepsy, generalized atonic	Focal epilepsy, generalized atonic
Other diagnoses		LGS	LGS, ASD	ASD	LGS	Tuberous sclerosis, LGS, ASD	Pontocerebellar hypoplasia, static encephalopathy		LGS	Anoxic brain injury	LGS	(Case 10)
Prior ASM usage		Clobazam, zonisamide	Levetiracetam, oxcarbazepine, lamotrigine, phenytoin	Lamotrigine	Felbamate, ethosuximide, valproic acid, clobazam	Levetiracetam, lamotrigine, brivaracetam, phenobarbital	Levetiracetam, oxcarbazepine, rufinamide	Levetiracetam, oxcarbazepine, midazolam	Levetiracetam, cannabidiol. rufinamide, diazepam	Clonazepam, topiramate, levetiracetam, phenobarbital, oxcarbazepine, lamotrigine, clobazam	Levetiracetam, lamotrigine, clobazam, clonazepam	Levetiracetam, lamotrigine, clobazam, clonazepam

Abbreviations: ASD, autism spectrum disorder; ASM, anti‐seizure medications; LGS, Lennox Gastaut syndrome.

### 3.2 Characteristics of MRgLITT callosotomy

MRgLITT callosotomy was pursued for the treatment of drop attacks in 10 cases and for generalized absence and myoclonic seizures in one case. Three patients were consulted for completion of prior open callosotomy, 3‐, 4‐, and 18‐month post‐microsurgical anterior two‐thirds (n = 2) callosotomy, respectively, or complete callosotomy (n = 1). One of these patients underwent MRgLITT again for completion of callosotomy seven months later, including ablation of genu and residual splenium, with simultaneous responsive neurostimulation (RNS) implantation within the centromedian nucleus. The remaining patients were prior callosotomy naïve and underwent anterior two‐thirds (n = 2), isthmus and genu (n = 1), or complete callosotomy (n = 4).

Four patients underwent concurrent placement of vagus nerve stimulator (VNS) during their MRgLITT callosotomy and four patients had VNS at the time of their prior open callosotomy. Decision to implant VNS in the same operation was based on discussions at multidisciplinary seizure conference in the case that callosotomy was not anticipated to treat the whole of patient's drug‐resistant seizure types. Generally, if the VNS is not MR‐safe at the MRgLITT field strength or the placement of the MR‐safe device falls outside the manufacture's guidelines, then it is not possible to pursue MRgLITT. If the patient underwent MR‐safe VNS placement previously, a chest x‐ray was acquired pre‐operatively to confirm placement prior to surgery. In the event patients had to undergo chest x‐ray on the same day of MRgLITT, additional time was budgeted to acquire clearance by radiology pre‐operatively. VNS was placed under the same anesthesia following MRgLITT ablation. Postoperatively, chest X‐ray was acquired to confirm VNS lead positioning.

Complete MRgLITT callosotomy (N = 4) was performed with 3‐4 trajectories, while anterior two‐thirds (N = 2) was performed with two to three trajectories and posterior completion (N = 2) was performed with two trajectories. Median cumulative laser on time 10.7 (IQR 5.6‐15.0) min and was directly related to the number of trajectories used. Total ablation volume averaged 5.5 ± 2.3 cm^3^. Surgery length ranged from 4 to 8 hours (5.8 ± 1.7), with blood loss of 6±5mL, and total anesthesia time of 7.8 ± 1.5 hours (see Table 2).

**TABLE 2:** Perioperative characteristics

	Summary	Case 1	Case 2	Case 3	Case 4	Case 5	Case 6	Case 7	Case 8	Case 9	Case 10	Case 11
MRgLITT procedure		Complete CC	Posterior 1/3 CC	Ant 2/3 CC	Complete CC	Complete CC	Complete CC	Ant 2/3 CC	Complete CC	Genu & splenium	Posterior 1/3 CC	Residual splenium w/ RNS placement
Prior surgery			Microsurgical ant 2/3 CC; VNS placement						Microsurgical CC; VNS placement		Microsurgical ant 2/3 CC; VNS placement	(Case 10)
VNS, n (%)	6 (75)	Yes	Yes	Yes	Yes	Yes			Yes	Yes	Yes	Yes
LITT trajectories, M (SD)	3	3	2	2	3	4	3	3	2	4	2	1
Laser on time (min), M (SD)	12.4 ± 7	10.9	5.6	20.8	8.3	24.4	13.6	10.4	3.72	14.95	4.8	2.57
Surgery length (h), M (SD)	5.8 ± 1.7	5.6	2.9	6.9	6.8	8.1	3.9	6.4	7.13	9.03	5.8	7.18
Blood loss (mL), M	6 ± 5	5	5	10	10	15	5	0	0	5	0	50
LOS ICU (d), median (IQR)	1 (1‐2)	2	1	2	1	5	2	1	1	1	1	3
LOS floor (d), median (IQR)	1 (0‐1)	1	0	1	1	1	1	0	0	1	1	0
Total LOS (d), median (IQR)	2 (1‐3)	3	1	3	2	6	3	1	1	2	2	3
Discharge home, n (%)	6 (82)	Rehab	Home	Home	Home	Rehab	Home	Home	Home	Home	Home	Home
Postoperative transient functional change, n (%)	4 (36)	Left‐sided weakness; back to baseline after rehab	None	None	None	Worsened truncal ataxia; back to baseline after rehab	Weakness, difficulty ambulating; baseline at discharge	Non‐verbal 6 d post‐op, self‐resolved	None	None	None	None
Permanent neurologic deficit	0	None	None	None	None	None	None	None	None	None	None	None

Abbreviations: CC, corpus callosotomy; VNS, vagal nerve stimulatory; RNS, responsive nerve stimulator.

### 3.3 Hospital course

Anti‐seizure medications were continued in the perioperative period for all patients. Patients were discharged after a median of two days (IQR 1‐3), including median one day (IQR 1‐2) in the ICU and one day (IQR 0‐1) on the floor. Eight patients were discharged at neurological baseline, and two patients were discharged to inpatient rehab. No patients required readmission. All patients were treated with a course of dexamethasone with 1‐2 week taper (see Table 2).

### 3.4 Outcomes

Follow‐up available at the time of data entry averaged 8.6 ± 6.9 months. Of 11 total cases, 10 underwent callosotomy for drop attacks, seven of whom had at least 6 months of follow‐up. Of these seven, 71% (N = 5) had resolution of drop attacks at longest follow‐up. The other two patients had not maintained seizure freedom. Of the four cases with <3 months of follow‐up at the time of data collection, two have experienced a significant reduction in drop attacks, one has remained free of drop attacks, and one has not experienced any improvement (see Table 3). Two callosotomy naïve patients that underwent anterior two‐thirds callosotomy have not experienced reduction in drop attacks. The four callosotomy naïve patients who had complete callosotomy experienced complete resolution within the first 6 months.

**TABLE 3:** Patient seizure outcomes

	Summary	Case 1	Case 2	Case 3	Case 4	Case 5	Case 6	Case 7	Case 8	Case 9	Case 10	Case 11*
Total follow‐up (months)	8.6 ± 6.9	14.96	10.65	17.98	11.84	13.60	0.73	0.50	5.51	1.82	16.39	0.50
Atonic seizures												
Pre‐op seizures	7 ± 6/d	10‐15/d	1‐3/d	NA	10‐25/d	1‐20/d	4‐7/d	3‐6/d	10	NA	1/d	1‐3/d
Post‐op seizures	4 ± 7 /wk	0	0	NA	0	0	0	3‐6/d	0	NA	1/d	0‐1/d
Other seizures												
Pre‐op seizures	5 ± 9/d	20‐30/d	1‐3/day	1‐2/ wk	5‐8/d	1/d	3/wk	NA	1‐5/d	1‐100/d	2‐3/mo	30/mo
Post‐op seizures	1 ± 2/d	2‐5/d	8‐10/wk	1‐2/ wk	2‐3/d	0	0	NA	2‐3/d	3‐5/d	2‐3/mo	0
Engel Class	70% improved	IV	II	IV	II	III	II	NA	III	III	IV	I

Abbreviation: NA, not applicable.

* Repeat patient, original procedure is Case 10.

**FIGURE 2:**
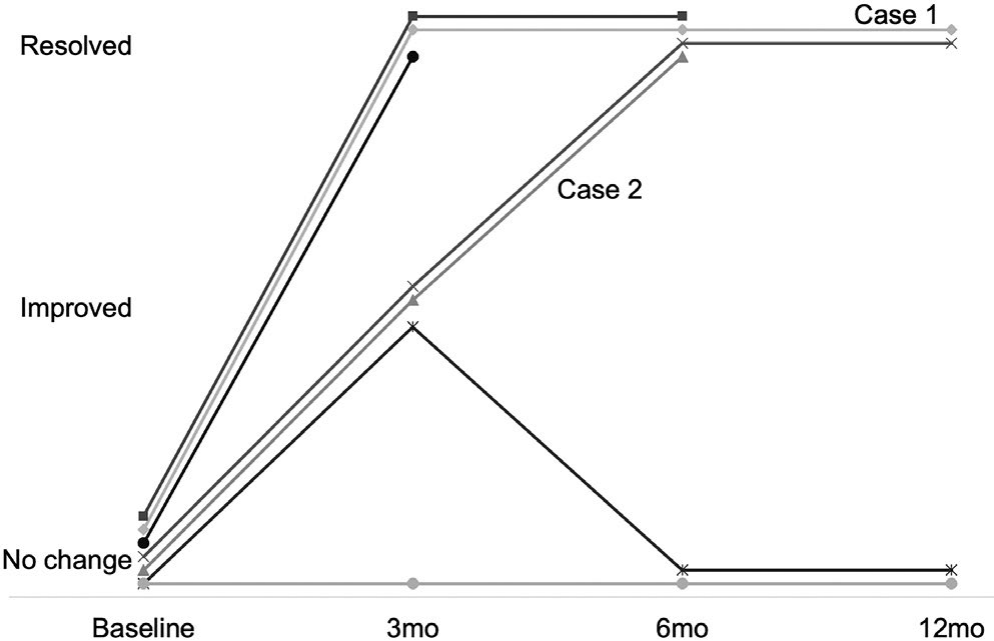
Timeline of postoperative changes in seizure frequency for the 7 patients with follow‐up data. “Improved” represents at least 50% reduction of baseline seizure frequency at the follow‐up timepoint. “Resolved” represents complete freedom from atonic seizures

Following MRgLITT corpus callosotomy, 43% (n = 3/7) were experiencing no drop attacks at 3‐month follow‐up and 86% (n = 6/7) had experienced improvement. At 6‐month follow‐up, 83% of cases (n = 5/7) were free from drop attacks, while the other 17% had not improved. At 12‐month follow‐up, 67% were free from drop attacks (n = 4/6) and 33% had returned to or remained at baseline (see Figure 2).

In addition to mitigation of drop attacks, MRgLITT callosotomy resulted in an improvement of other seizure semiologies in 57% of cases with at least 3‐month follow‐up (n = 4/7), including generalized myoclonic, generalized tonic‐clonic, and focal seizures (Engel Class II: 28%, Class III: 28%, Class IV: 43%). The patient for whom LITT callosotomy was performed for absence and generalized myoclonic seizures did not experience improvement in their seizure frequency (see Table 3).

### 3.5 Complications

No intraoperative complications were encountered in this series. One patient had a small asymptomatic intraventricular hemorrhage on postoperative scan that remained stable on follow‐up scans. Postoperatively, four patients experienced transient disconnection syndromes. Three patients experienced impaired mobility and coordination, and one patient was found to have impaired ability to vocalize and visually track objects postoperatively. Two of these patients, who notably had undergone complete callosotomy, received inpatient therapy for 2 and 3 weeks, respectively, for the treatment of their disconnection syndrome, while the other two patients improved at discharge and continued to improve with outpatient physical therapy. Deficits resolved at postoperative follow‐up in all affected patients.

### 3.6 Illustrative cases

#### 3.6.1 Case 1

A 6‐year‐old, right‐handed girl diagnosed with epilepsy at the age of 2 years, who initially presented with febrile status epilepticus. This patient's seizures remained resistant to multiple anti‐seizure medications, including zonisamide, clobazam, levetiracetam, and lamotrigine, and had previously failed trials of ethosuximide. At the time of evaluation, she was experiencing 20 to 30 seizures per day, each lasting 1‐2 minutes in length, including atypical absence and 10 to 15 drop attacks per day. Phase 1 evaluation for epilepsy surgery demonstrated persistent generalized spike and wave discharges, occasional independent right and left frontal and occasional spike wave discharges, background slowing, and disorganization.

**FIGURE 3:**
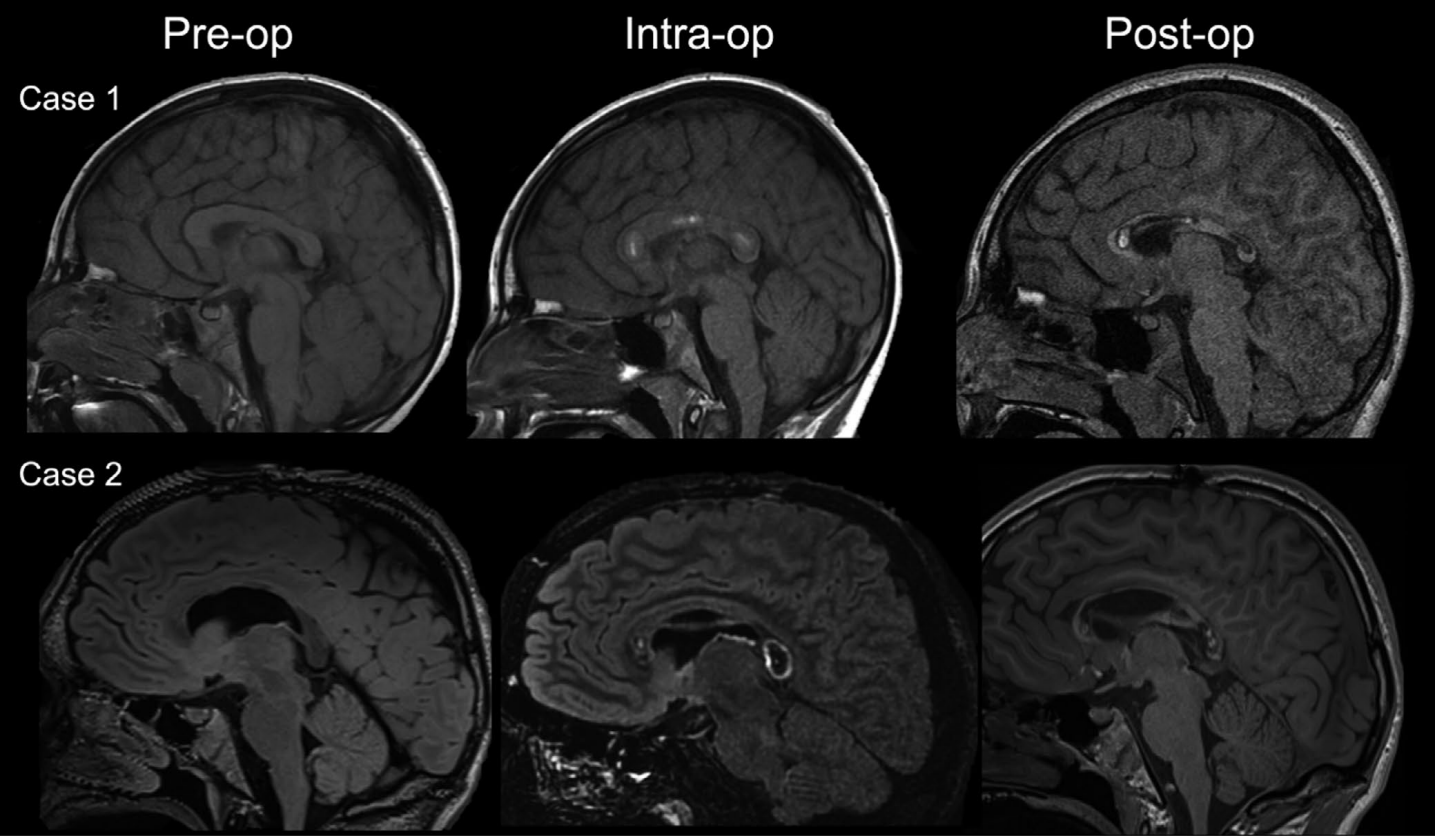
Representative imaging of stable procedural changes of the corpus callosum, before, during, and after MRgLITT complete callosotomy (Case 1) and ablation of the genu for the completion of prior anterior two‐thirds open callosotomy (Case 2)

She underwent an uncomplicated MRgLITT callosotomy with left‐sided VNS placement at 7 years of age. Three LITT trajectories were utilized to ablate the splenium, genu, and body of the corpus callosum (Figure 3). She tolerated the procedure well. She was discharged to a rehab facility on postoperative day 3 for functional deficits including impaired mobility, balance and coordination, and dysphagia attributed to mild, postoperative left‐sided weakness. This improved over 2 weeks of inpatient rehab, and patient was taking all food and medications per os at discharge. Postoperative MRI demonstrated expected postoperative changes in the corpus callosum. She was discharged on a 7‐day dexamethasone taper.

The patient was free from drop attacks at 3‐month follow‐up, and her absence seizures were found to have significantly decreased in duration and frequency to less than ten per day. She remained free of drop attacks at 6 months, but her epilepsy had evolved to include daily focal seizures with impaired awareness. Her lamotrigine was increased, and VNS settings were adjusted. This patient's deficits had resolved by 6 months, and she experienced no additional complications during her follow‐up.

#### 3.6.2 Case 2

A 15‐year‐old boy with drug‐resistant mixed epilepsy diagnosed at 2 years, consistent with Lennox‐Gastaut syndrome. Baseline seizure semiology included focal seizures with impaired awareness with and without generalization to bilateral tonic‐clonic seizures, as well as drop attacks, followed by left postictal paralysis. At the time of presentation, this patient continued to have between one and three seizures per day, despite multiple anti‐seizure medications, including lamotrigine and levetiracetam. He had previously failed trials of zonisamide, oxcarbazepine, clonazepam, and divalproex. Due to the severity of his seizures and inability to cooperate with prolonged Phase 1 evaluation for epilepsy surgery, he underwent an open corpus callosotomy (anterior two‐thirds) and vagal nerve stimulator placement at 15 years of age. MRI at 3‐month follow‐up revealed a small area of residual genu and remaining splenium of the corpus callosum.

While his seizures did gradually improve to around five per week, he continued to experience injurious drop attacks. Completion callosotomy was pursued 4 months later using MRgLITT to ablate the splenium and remaining genu (see Figure 3). This patient did not experience any complications postoperatively and was discharged home on postoperative day one on a 10‐day taper of dexamethasone.

At 3‐month follow‐up, he was experiencing fewer than 10 generalized tonic‐clonic seizures monthly and no drop attacks. Postoperative imaging showed small area of residual connection of the splenium of unclear clinical significance. The patient remained free from drop attacks 5 months later, and postictal paralysis had improved to 5‐15 minutes (previously 30‐40 minutes).

## 4 DISCUSSION

This study describes experience performing MRgLITT callosotomy in a series 11 cases, within 10 patients, to demonstrate its safety in pediatric populations. In a heterogeneous sample of completion posterior, anterior two‐thirds, total callosotomy, and residual callosal fiber ablation, we found excellent results with respect to drop attacks, as well as zero percent intraoperative complication rate and zero persistent functional deficits at follow‐up. At last follow‐up, 71% of patients with at least 6‐month follow‐up were free from drop attacks, without long‐term neurological deficits. We envision that MRgLITT callosotomy may serve a complementary role to open callosotomy, where MRgLITT would be the treatment of choice for medically complex patients or for those with prior surgery. Our technique is equally suitable as a salvage procedure after failed open (or in theory, MRgLITT) callosotomy.

Literature describing MRgLITT callosotomy in pediatric patients consists of few case series, though continues to grow rapidly as multiple centers develop this technique. Since initial case reports by Ho and colleagues in 2016^15^ and Karsy and co‐authors in 2018,^17^ multiple studies have further detailed operative technique and results.^1^, ^4^, ^6^, ^7^, ^8^, ^9^ Caruso and colleagues recently reported MRgLITT callosotomy in six pediatric patients with eight procedures total.^11^ Their report used a similar technique reported here, including 3‐4 trajectories, operative time, length of stay, and blood loss. Although Caruso does not discuss seizure freedom rates, they report patients had good response overall to MRgLITT callosotomy. In another series, Roland et al reported LITT anterior two‐thirds callosotomy in a series of 10 patients with 1‐3 trajectories, of which 20% had excellent responses in targeted seizure control and an additional 30% had significant responses.^24^ Our results align with previous reports of open callosotomy, adding to the growing body of literature, suggesting MRgLITT callosotomy may indeed be equivalent to open callosotomy.^1^, ^4^, ^6^, ^7^, ^8^, ^9^

There are multiple factors that influence the success of open callosotomy, including younger age at surgery (<12 years) and intractable epilepsy characterized by drop attacks.^1^, ^2^ Younger patients may experience better functional outcomes after callosotomy, given the privileged ability of neural plasticity to rewire networks disturbed by callosotomy.^25^ We anticipate that these factors would also affect the success of MRgLITT callosotomy, although a larger population is required to make such conclusions. One precaution that must be acknowledged is that response to callosotomy (either open or MRgLITT) may decline over time. Only 35% of patients with drop attacks were seizure free at 5 years in a large meta‐analysis of open callosotomy outcomes.^26^

MRgLITT technique leverages live imaging in which the degree of ablation can be confirmed immediately with T1‐weighted or FLAIR images.^27^ Postoperative imaging in multiple patients in this study suggested that there may have been small amounts of residual tissue in the corpus callosum, even in patients with good clinical outcomes. While remaining fibers may be visualized acutely, others may become more obvious at 3‐month follow‐up. It remains unclear whether this tissue consists of functional fibers or gliotic tissue and further study is warranted to elucidate the role of this residual tissue in seizure freedom. Others, including Huang et al, have found similar results.^16^ Repeat MRgLITT ablation may be used to ablate remaining fibers if clinically indicated.

Limitations of this report include its retrospective nature and limited follow‐up window. Selection bias during the evaluation process may limit the generalizability of the results. This report emphasizes the safety of MRgLITT in pediatric populations and presents preliminary seizure outcomes. Additionally, four patients had VNS placed at the time of callosotomy, which may confound the improvement in non‐atonic seizure types. Further large‐scale and multi‐center trials are required to draw definitive conclusions.

## 5 CONCLUSION

MRgLITT corpus callosotomy is safe and effective modality for the management of drug‐resistant epilepsy in the pediatric population, especially drop attacks. The indications and results for MRgLITT callosotomy are comparable to open callosotomy, but MRgLITT‐based techniques offer the possibility of shorter hospitalization, fewer patient activity restrictions, and less surgical morbidity. This technique can be utilized for total callosotomy, anterior two‐thirds callosotomy, and posterior one‐third completion callosotomy. Further studies are required to generalize these results and delineate the safety and efficacy of MRgLITT callosotomy compared to open techniques.

## CONFLICTS OF INTEREST

Dr Abel is a consultant for Monteris Medical and receives research funding through Monteris Medical for the LAANTERN Trial. Dr Mallela, Dr Abou‐Al‐Shaar, Ms Hect, and Ms Akwayena do not have conflicts of interest to disclose.

## ETHICAL PUBLICATION

We confirm that we have read the Journal's position on issues involved in ethical publication and affirm that this report is consistent with those guidelines.
